# A case-control study of determinants for high and low dental caries prevalence in Nevada youth

**DOI:** 10.1186/1472-6831-10-24

**Published:** 2010-11-11

**Authors:** Marcia Ditmyer, Georgia Dounis, Connie Mobley, Eli Schwarz

**Affiliations:** 1 UNLV School of Dental Medicine, 1001 Shadow Lane, MS 7425, Las Vegas, NV 89106, USA; 2 University of Sydney, Faculty of Dentistry and UNLV School of Dental Medicine, 1001 Shadow Lane, MS 7425, Las Vegas, NV 89106, USA

## Abstract

**Background:**

The main purpose of this study was to compare the 30% of Nevada Youth who presented with the highest Decayed Missing and Filled Teeth (DMFT) index to a cohort who were caries free and to national NHANES data. Secondly, to explore the factors associated with higher caries prevalence in those with the highest DMFT scores compared to the caries-free group.

**Methods:**

Over 4000 adolescents between ages 12 and 19 (Case Group: N = 2124; Control Group: N = 2045) received oral health screenings conducted in public/private middle and high schools in Nevada in 2008/2009 academic year. Caries prevalence was computed (Untreated decay scores [D-Score] and DMFT scores) for the 30% of Nevada Youth who presented with the highest DMFT score (case group) and compared to the control group (caries-free) and to national averages. Bivariate and multivariate logistic regression was used to analyze the relationship between selected variables and caries prevalence.

**Results:**

A majority of the sample was non-Hispanic (62%), non-smokers (80%), and had dental insurance (70%). With the exception of gender, significant differences in mean D-scores were found in seven of the eight variables. All variables produced significant differences between the case and control groups in mean DMFT Scores. With the exception of smoking status, there were significant differences in seven of the eight variables in the bivariate logistic regression. All of the independent variables remained in the multivariate logistic regression model contributing significantly to over 40% of the variation in the increased DMFT status. The strongest predictors for the high DMFT status were racial background, age, fluoridated community, and applied sealants respectively. Gender, second hand smoke, insurance status, and tobacco use were significant, but to a lesser extent.

**Conclusions:**

Findings from this study will aid in creating educational programs and other primary and secondary interventions to help promote oral health for Nevada youth, especially focusing on the subgroup that presents with the highest mean DMFT scores.

## Background

By the year 2000, the World Health Organization (WHO) announced the global average goal for dental caries was to be no more than 3 DMFT (decayed, missing, filled teeth) at 12 years of age [1]. Although there has been a significant decline in dental caries prevalence since the early 1970s, oral disease, including caries, remains a major public health challenge [2-4]. In 2004, most American children reported good oral health, but subsets suffered a higher level of oral disease, primarily children living in poverty and some racial/ethnic minority populations [5]. American children born into poverty have suffered twice as much tooth decay as their more affluent peers and have likely had less access to oral health care [5]. Problems with access to health care result in uninsured children being 2.5 times less likely than insured children to receive dental care and 3 times more likely than insured children to have unmet dental needs [5].

The decayed, missing, and filled teeth (DMFT) index is commonly accepted by the dental community for measuring caries prevalence in the population and has been used repeatedly in the National Health and Nutrition Examination Survey (NHANES) [4]. Over time, a skewed distribution of caries prevalence has developed in many countries, with a significant proportion of 12-year-olds found with high or very high DMFT values even though a proportion is totally caries free [6]. Some suggest that mean DMFT does not accurately reflect this skewed distribution leading to incorrect conclusions that the caries situation for the whole population is under control, while in reality population subgroups still suffer from high caries rates. WHO reported that oral disease, including dental caries, remains a major public health challenge [2]. In the USA, dental caries is the single most common childhood disease, occurring 5 times more frequently than asthma and 7 times more than hay fever [3].

Studies have identified associations between numerous factors and dental caries, supporting agreement that dental caries is a multi-factorial disease modulated by genetics, behavior, and environment [2,7]. Understanding the influence of demographic variables, such as sex, race, community water fluoridation, environmental smoke, and lifestyle and social conditions will contribute to the development of improved prevention and treatment approaches [2,4-6]. Thus, identifying the subpopulations at greatest risk (those presenting with the highest DMFT scores) and discovering the significant correlations of selected factors associated with dental caries prevalence provides the rationale for this study.

The purpose of this study was two-fold. Firstly, to identify the 30% of Nevada Youth assessed during a statewide, school-based oral health screening initiative who presented with the highest DMFT score and compare it with a cohort who were caries free and with comparable national NHANES data. Secondly, to explore the factors associated with caries prevalence among those who presented with the highest DMFT scores compared to the caries-free group.

## Methods

### Selection and Description of Participants

Since 2001, an ongoing statewide, school-based, oral health screening initiative annually has been conducted in public/private middle and high schools in Nevada. Data used for this retrospective case/control study collected during 2008/2009 academic school year included 4169 adolescents between ages 12 and 19 (Case Group: n = 2124); Control Group: n = 2045). Inclusion criteria for participation in the health screenings were parental consent and student assent. The data was reorganized in ascending order starting with a DMFT index = 0. The entire sample was subdivided into three equal segments, from which those who are caries free (DMFT = 0) would constitute the control group and those in the top 1/3 with the highest DMFT (DMFT ≥ 4.0) would constitute the case group. The third that fell in the middle (DMFT < 0 > 4.0) were not used for the purposes of this study. The University of Nevada Las Vegas Institutional Review Board approved this initiative to assure student confidentiality.

### Oral Health Screening

Trained and calibrated licensed dental examiners conducted oral health screenings in dedicated mobile dental clinics (one each in northern and southern Nevada) to assess caries prevalence. Inter- and intra-examiner reliability were validated with Intraclass Correlation Coefficients (0.81; p < 0.001 and 0.98; p < 0.001 respectively) [8].

Examiners followed the Radike criteria with modifications to establish prevalence [9]. As with NHANES, prevalence was determined using DMFT indices developed by Klein et al [10] and prevalence of untreated tooth decay. Examiners used artificial light and non-magnifying mirrors to perform visual assessments similar to methods used in NHANES [4].

The *Crackdown on Cancer *oral screening initiative procedural manual (contact authors) detailed all training/calibration, diagnostic, and coding criteria.

### Face-to-Face Interviews

Trained interviewers collected self-reported health behaviors, health history, and environmental determinants associated with disease through face-to-face interviews in the privacy of the mobile clinic setting. Investigators computed the internal reliability of the questionnaire using Cronbach's alpha (r = 0.79, p < 0.001) [11].

### Selection of Variables

Eight factors comprised the cited variables of interest in the dental literature as significant modulators of dental caries [1,4,12]. These included sex, age, dental insurance status, race, environmental smoke exposure, smoking habits (including cigarettes, cigars, smokeless tobacco, and marijuana), living in an area with or without community water fluoridation, and applied dental sealants.

In 1999, the Nevada Legislature passed a bill requiring the Southern Nevada Water Authority to fluoridate the municipal water supply [12-14], subsequently establishing the fluoride range of 0.7-1.2 mg/L for Clark County [12]. Clark County, which comprises around ¾ of the population of Nevada, is the only county in Nevada with community water fluoridation. Consequently, the exposed group comprised students attending schools in Clark County versus those attending schools in all other areas of Nevada.

### Statistical Analysis

Caries prevalence rates were computed (untreated decay scores {D-Scores} and DMFT scores) for the 30% of Nevada Youth who presented with the highest DMFT score and compared to the control group (caries-free) as well as to data from NHANES (1999-2004) [4]. Computed t-tests and Analysis of Variance (ANOVA) compared D-Score means and DMFT score means on select variables of the case group. Bonferroni post hoc tests were used to assess significant differences between groups [11].

Chi-Square analyses were computed for each variable to assess whether significant differences were found between the case and control groups. Bivariate logistic regression was subsequently performed (forward stepwise) to assess relative contributions of these predictor variables between the case and control groups. Logistic regression was used because it does not assume that the relationship between the independent variables and the dependent variable is a linear one, nor does it assume that the dependent variable or the error terms are distributed normally. The Wald statistic was used to assess statistical significance. Odds Ratios (OR) associated with each predictor value were produced. There was no presence of multicollinearity among the significant variables [11]. Subsequently, multivariate logistic regression was performed using backwards stepwise methods to calculate OR (Referent: DMFT = 0) for each of the eight variables using the Wald statistic to confirm significance of each variable and Chi-Square to validate the model's goodness-of-fit (χ^2 ^= 475.35, p = 0.002). Data reported in this study were analyzed using SPSS 16.0 (SPSS, Inc.).

## Results

This study included an equal proportion of males and females (49% male; 51% female), with an approximately equal proportion split between areas with community water fluoridation (46%) and those without community water fluoridation (54%). A majority of the sample was non-Hispanic (62%), non-smokers (80%), and with dental insurance (70%). Table 1 details the demographics between the case and control groups within all select variables. Table 1 also details the mean untreated decay scores (D-Score) and mean DMFT scores of the case group by select variables.

**Table 1:** Control- and case Group Demographics and Mean D and DMFT Scores of the Case Group

Variable	Control Group	Case Group	D-Score†	Test Statistic	DMFT†	Test Statistic
	N	N	Mean (SE)	D-Score	Mean (SE)	DMFT
Sex						
Male	1086	953	2.84 (0.10)	1.41	6.36 (0.14)	11.222**
Female	959	1171	3.04 (0.09)		7.01 (0.04)	
Age‡						
12-15	1654	1346	2.77 (0.08)	3.06*	6.66 (0.09)	31.250**
16-19	388	781	3.21 (0.12)		7.45 (0.07)	
Dental Insurance						
Insured	1176	1353	2.14 (0.8)	15.18**	6.87 (0.07)	19.89**
Not Insured	383	678	4.79 (0.12)		8.03 (0.06)	
Race Group‡						
White, Non-Hispanic	1296	842	2.19 (0.10)±	10.13**	6.86 (0.08)	13.89**
Black, Non-Hispanic	173	278	2.99 (0.12)		6.68 (0.12)	
Hispanic	573	1007	3.16 (0.11)		7.14 (0.10)	
Fluoridation						
Fluoridation	1324	580	1.72 (0.07)	15.55**	6.43 (0.08)	14.20**
Without Fluoridation	721	1544	3.78 (0.09)		7.30 (0.07)	
Second Hand Smoke						
Exposed	565	871	3.52 (0.11)	7.13*	7.23 (0.06)	6.09*
Non-exposed	1470	1244	2.03 (0.09)		6.74 (0.10)	
Smoking Status						
Currently Smoke	215	372	3.53 (0.14)	5.03*	7.43 (0.12)	23.65**
Currently do not Smoke	1177	1204	2.60 (0.09)		6.33 (0.05)	
Applied Sealants						
No Sealants	752	1153	3.63 (0.10)	11.29**	7.36 (0.12)	7.69*
Sealants	1290	974	2.11 (0.08)		6.45 (0.14)	

Note: *p < 0.05; ** p < 0.01; †D-Score and DMFT Scores computed on the 30% with the highest DMFT Scores; ‡ Groups as Defined in NHANES, 2007;

± Bonferroni post hoc results indicate differences between this group and the other 2 groups.

Table 2 compares sex, age group, and race groups of the case group to population data reported in NHANES 1999-2004 [4]. Only the 30% of Nevada adolescents with the highest mean DMFT were compared. A review of the D-Scores and DMFT Indices of this subpopulation reflected a much higher percentage within each comparison group. These data confirmed a skewed distribution of dental caries in this subpopulation.

**Table 2:** Comparison of the 30% Adolescents with the Highest DMFT Score from Nevada Oral Health Initiative with NHANES Population Data (1999-2004)

30% with the Highest DMFT Score*			NHANES (1999-2004)		
**Variable**	Decay** % (SE)	**DMFT Mean (SE)**	**Variable**	Decay** % (SE)	**DMFT Mean (SE)**

**Age**			**Age**		
12-15	28.7% (1.21)	6.66 (0.09)	12-15	16.91% (0.99)	1.78 (0.08)
16-19	45.3% (1.11)	7.45 (0.07)	16-19	22.24% (1.45)	3.31 (0.09)
**Sex**			**Sex**		
Males	46.2% (1.13)	6.36 (0.14)	Males	19.89% (1.22)	2.31 (0.09)
Females	53.8% (1.21)	7.01 (0.04)	Females	19.31% (1.30)	2.79 (0.08)
Race/Eth*			**Race/Eth**		
White, NH	26.3% (1.46)	6.86 (0.08)	White, NH	16.22% (1.45)	2.54 (0.10)
Black, NH	37.0% (1.44)	6.68 (0.12)	Black, NH	25.66% (1.39)	2.20 (0.10)
Hispanic	41.8% (1.22)	7.14 (0.10)	Hispanic	28.57% (1.54)	2.82 (0.13)

Note. * N = 2127 **Untreated Caries; NHANES, 2007 estimates are adjusted to the US 2000 standard population; SE = Standard Error.

Significant differences in mean D-scores (untreated decay) were found in seven of the eight variables in the case group (Table 1). The only variable with no significant difference in the mean D-Score was sex. There were significant differences between the case and control groups in mean DMFT Scores on all eight predictor variables.

Chi-Square analysis revealed significant differences between the case and controls in all selected variables (Table 3). The Wald statistic confirmed significance in seven of the eight variables in the bivariate logistic regression between the case and control groups (Table 3). There were no significant differences found in those who smoked and those who did not smoke between the case and control groups. Chi-Square validated the model's goodness-of-fit (χ^2 ^= 172.04, p = 0.0009). Odds ratios produced the relative odds of the groups within each factor between the case and controls (Table 3). The strongest contributors were race group (F = 152.78, p < 0.001), increasing age (F = 58.40, p < 0.001), community water fluoridation (F = 57.73, p < 0.001), and applied sealants (F = 52.88, p < 0.001). Odds ratios found that Hispanics with the highest reported mean DMFT scores were more than 2 times (OR = 2.135, CI = 0.29-0.59) more likely than White, Non-Hispanics to have the highest reported mean DMFT. Similarly, Nevada youth between 16 and 19 years of age were more than 2 times (OR = 2.04, CI = 0.41 - 0.59) more likely than those between age 12-15 to be among the adolescents with the highest DMFT score. Those living in areas where there is no community water fluoridation were almost 2 times (OR = 1.98, CI = 0.40-0.59) more likely than those living in communities with fluoridated municipal water supply to be among the highest DMFT scores. Those without sealants are 1 1/2 times (OR = 1.52, CI = 1.59 - 2.25) more likely than those with sealants to present with the highest mean DMFT.

**Table 3:** Results of Chi-Square tests on Select Variables and Bivariate Logistic Regression of Significant Variables from Chi-Square Tests

Variable	Control Group		Case Group		**Χ**^**2 **^**Statistics**	Caries Free vs. Top 30% DMFT Score		Wald Statistic
	N	%	N	%		OR	(95% CI)	
**Sex**								21.25*
Male	1086	53.1	953	44.9	27.64*	1.00		
Female	959	46.9	1171	55.1		1.25	(1.09-1.41)	
**Age‡**								58.40***
12-15	1654	81.0	1346	63.3	161.06**	1.00		
16-19	388	19.0	781	36.7		2.04	(1.75 - 2.33)	
**Dental Insurance**								46.59*
Insured	1176	75.4	1353	66.6	63.13**	1.00		
Not Insured	383	24.6	678	33.4		1.50	(1.25 - 1.75)	
**Race Group‡**								152.78***
White, Non-								
Hispanic †	1296	63.5	842	39.6				
Black, Non-					240.08**	1.00		
Hispanic	173	8.5	278	13.1		1.75	(0.82 - 2.98)	
Hispanic †	573	28.0	1007	47.3		2.35	(2.05 - 2.55)	
**Fluoridation**								57.73***
Fluoridation	1324	64.7	580	27.3		1.00		
Without					35.21*			
Fluoridation	721	35.3	1544	72.7		1.98	(1.40 - 2.56)	
**Second Hand Smoke**	565	27.8	871	41.2		1.00		32.38*
Exposed	1470	72.2	1244	58.8	85.41**	1.33		
Non-exposed							(1.08 - 1.58)	
**Smoking Status**								2.05
								
Currently Smoke	215	15.4	372	23.6	31.01*	1.00		
Currently do not Smoke	1177	84.6	1204	76.4		1.16	(0.94-1.38)	
**Applied Sealants**								52.88***
No Sealants	752	36.8	1153	54.2		1.00		
Sealants	1290	63.2	974	45.8	126.13**	1.52	(1.39-1.65)	

Note: *p < 0.05; ** p < 0.01; ***p < 0.001; D-Score and DMFT Scores computed on the 30% with the highest DMFT Scores; ‡ Groups as Defined in NHANES, 2007; †Significant differences between 2 groups only

The relative strength of the variables selected was further explored using multivariate logistic regression analysis (Table 4). All eight variables contributed significantly to the final model (F = 458.93, p < 0.001) R^2 ^= 0.443). The R^2 ^of 0.443 (Adjusted R^2 ^= 0.412) indicated approximately 40% of the variables combined contributed to increased DMFT indices. Beta coefficients placed race, age, fluoridation, and applied sealants as the strongest contributors, respectively. Gender, second hand smoke, insurance status, and tobacco were significant, but to a lesser extent.

**Table 4:** Multiple Logistic Regression Results Demonstrating Odds-ratios of Being in the High DMFT Group vs. in the Cariesfree Group.

		Caries Free vs. Top 30% DMFT Score	
		
Variable	Wald Statistic	OR	(95% CI)
**Race Group**	159.63*		
White, Non-Hispanic		1.00	
Black, Non-Hispanic		2.27	(2.37 - 3.37)
Hispanic		2.96	(2.05 - 3.27)
**Age**	59.784***		
12-15		1.00	
16-19		2.04	(1.70 - 2.45)
**Fluoridation**	59.233***		
Fluoridation		1.00	
Without Fluoridation		2.04	(1.69 - 2.45)
**Applied Sealants**	53.833***		
Sealants		1.00	
No Sealants		1.53	(1.44 - 1.63)
**Second Hand Smoke**	33.1258***		
Non-exposed		1.00	
Exposed		1.42	(1.03 - 1.53)
**Insurance Status**	6.587*		
Insured		1.00	
Not Insured		1.25	(1.04 - 1.51)
**Smoking Status**	6.479*		
Currently do not Smoke		1.00	
Currently Smoke		1.85	(1.68 - 2.06)

All significant Independent Variables Remain in Final Model.

*p < 0.05; ***p < 0.001

## Discussion

This study reported caries prevalence between 30% of Nevada youth with the highest DMFT and Nevada youth who are caries free. Both the mean untreated decay score (D-Score) and the mean DMFT index were computed. Results reflect a skewed distribution of caries among those with the highest mean D-Score and mean DMFT on all selected variables. This study confirmed that dental caries remains a common chronic disease among Nevada youth and is especially prevalent among a select subpopulation.

The decade-old global average goal for dental caries to be no more than 3 DMFT (decayed, missing, filled teeth) at 12 years of age [1] was an aspirational goal set by the WHO at a time where it looked like caries would explode in developing as well as developed countries [15]. In many countries the goal was achieved, but oftentimes was replaced by a polarized situation where some had no and some had a significant number of dental caries; thus, the case group presented with a significantly higher prevalence rate in mean DMFT in all select variables, and a greater mean DMFT and mean D-score than the national average (Table 2). Dental professionals should be aware of these factors when developing educational programs, and when designing and implementing prevention strategies

The logistic regression produced significance among all but one of the select variables, smoking status. The strongest contributor was race group. The Nevada Oral Health Screening Initiative recorded race and ethnicity separately. Race and ethnicity were combined into the same groups used in NHANES studies for subsequent comparison (Table 2). When comparing DMFT indices by ethnicity category (non-Hispanic White, non-Hispanic Black, and Hispanic) of the 30% of Nevada youth with the highest DMFT scores with national averages reported in NHANES, the prevalence of the 30% of Nevada youth with the highest DMFT were significantly higher. In both Nevada and national data, Non-Hispanic Blacks and Hispanics presented with higher rates respectively. African Americans and Hispanics were between 1.75 and 2.35 times more likely than non-Hispanic Whites to present with higher mean DMFT scores. Identified reasons for racial disparities in oral health include both microbiological and behavioral factors including income, education, and residence [16]. The percent increase in diversity within the Nevada population overall from April 1, 2000 to July 1, 2006 was 24.9% as compared to the percent increase in the US of 6.4% [17]. The larger than national average increase may explain the oral health disparity seen in these data between the various races in Nevada, but also presents a bigger challenge to the dental community to choose the correct preventive approach in such a diverse population.

Comparisons of age categories revealed higher mean DMFT indices in those between 15-19 years of age from those who were younger (12-15 years of age), indicating a significant increase with age [3]. Odds Ratios confirm the finding that those who were ages 16-19 were more than 2 times more likely than the younger age group to present with higher DMFT indices. Although age is not a modifiable factor, dental professionals should educate parents and adolescents of the importance of good oral hygiene practices.

Community water fluoridation has been documented as the most cost-effective, equitable, and safe community-based approach to improving oral health [18]. Participants living in areas without community water fluoridation in Nevada were almost 2 times more likely to present with higher DMFT indices. The benefits of water fluoridation are proportionally higher for people who do not have regular access to other sources of fluoride [18]. Therefore, dental professionals should counsel patients living in non-fluoridated geographic areas on the importance of using other sources of fluoride. It is of special significance that several futile attempts have been made in Nevada to introduce community water fluoridation to other counties, such as Washoe County, which comprises around 15% of the population.

Dental sealants help to reduce incidence of dental caries. Without dental sealants, caries prevalence and severity will likely continue to increase in children [19]. Nevada's successful sealant program has demonstrated decreases in prevalence rates that are greater than the national average. This study confirmed that Nevada youth who have not received dental sealants are 1 1/2 times more likely to present with higher mean DMFT scores than those with sealants [19].

Some suggest that inadequate dental care for children of low-income families is due, in part, to lack of dental insurance [20]. Participants without dental insurance were 1 1/2 times more likely to present with higher mean DMFT indices than those with dental insurance. Despite improvements in children's oral health through prevention, disparities still exist among subpopulations [20]. Lack of dental insurance could explain the negative oral health outcomes experienced by children and minorities [3,20].

Secondhand smoke exposure reportedly causes immediate harm [21]. Nonsmokers, living in a smoking environment, are at greatest risk for negative health effects from secondhand smoke exposure. Investigators reported an association between environmental tobacco and risk of dental caries among children; suggesting children exposed to secondhand smoke have significantly higher rates of dental caries [22]. Participants exposed to environmental smoke were 33% more likely to present with higher mean DMFT indices than those not exposed.

In a meta-analysis of more than 50 epidemiological studies, females had a higher prevalence and severity of dental caries than males [23]. For both genders, their untreated decay was around 40% of the DMFT index and no significant gender difference was found in the decayed component. The significantly higher DMFT index for females is taken as an indicator that females may have received more dental treatment expressed as higher M or F in the index, girls presented with 1.25 times greater likelihood than boys of presenting with a higher mean DMFT Score. Although, like race and age, sex is not a modifiable risk factor, dental professionals should target female patients for education and aggressive preventive measures [23].

Tobacco has been linked to poor oral health, including smoker's palate and melanosis, oral candidosis, dental caries, periodontal disease, and oral cancers [24]. In this study, the likelihood of presenting with higher mean DMFT scores was no greater between those adolescents who smoke and those who do not smoke. There was a very low number of adolescent participants who reported smoking (N = 215) compared to those who reported not smoking (N = 1177) in the case group. This factor combined with the expectedly brief period of smoking likely contributed to the non-significant results found with this factor.

### Limitations and Future Recommendations

Because this sample was examined using a modified protocol, data for this study may be an underestimate of caries prevalence and severity compared to NHANES [4]. Unlike NHANES, there were restrictions placed by the funding agency in our study preventing the use of compressed air and explorers. However, researchers compared studies using visual methods without probes and drying with studies using visual/tactile methods with explorers and compressed air and subsequently found that only in groups with low caries prevalence were statistical differences observed [25]. Additionally, there is an increasing amount of research that indicates the use of a dental explorer for assessing noncavitated incipient lesions may not be a best practice approach [26]. Some experts suggest that the use of an explorer for this purpose can penetrate the surface and convert a subsurface lesion to a frank cavity [27]. Additionally, there have been a number of false-positive diagnoses on occlusal surfaces thus indicating the value of the dental explorer is limited [9,26,27].

Self-reports warrant caution in interpreting data. However, quality control documentation supports data collection and entry protocols. In the Nevada Oral Health Initiative, individual students were not followed over time due to confidentiality issues, thus preventing longitudinal data collection. Analysis of cross sectional data across all years of the ongoing studies would strengthen these findings. Sources of fluoride other than community water fluoridation are not identified in this study. Inclusion of other potential sources of fluoride supplementation may influence these results. Data from the oral health screening initiative is likely to be an underestimate of similar data reported from the NHANES [3] because of differences in study measures discussed in this paper. For practical reasons the number of variables selected were limited, for instance SES was not measured directly, but the dental insurance variable functioned as a proxy for this, albeit not as detailed. The over 40% explanation of the variation of the dependent variable does attest to the relevance and importance of the independent variables chosen. Since this study focused on the two extreme groups in caries prevalence, the cariesfree and the high DMFT scorers, this study cannot be used to make inferences about the general DMFT status in Nevada youth or the ranking of Nevada youth oral health in a national context. We will conduct further studies to help assess these issues and the associations presented in this study.

## Conclusion

We found that a young person of Hispanic background, older age, living in a community without water fluoridation, and not having had sealants placed was at a significant higher probability of being in the high DMFT group compared to being cariesfree. Findings from this study will aid in creating educational programs and other primary and secondary interventions to help promote oral health for Nevada youth, especially in the subgroup that presents with the highest mean DMFT scores. Because dental professionals have frequent contact with adolescents and their parents/guardians, findings from this study can provide a guide for early detection, prevention, and treatment practices.

## Competing interests

The authors declare that they have no competing interests.

## Authors' contributions

MD, GD, and CM have been engaged in the multi-year development of the surveillance program, including developing the protocols and guidance for examiners, data collection as well as ongoing quality control. MD performed the statistical analysis in collaboration with ES. The paper was drafted by MD, ES, GD, and CM contributed to its completion. All authors have read and approved the final manuscript.

## Pre-publication history

The pre-publication history for this paper can be accessed here:

http://www.biomedcentral.com/1472-6831/10/24/prepub
